# Scrotal Ultrasound in the Infertile Male—A Practical Compass for the Urologist

**DOI:** 10.1590/S1677-5538.IBJU.2025.9920.1

**Published:** 2025-11-25

**Authors:** Sandro C. Esteves

**Affiliations:** 1 ANDROFERT Clínica de Andrologia e Reprodução Humana Campinas SP Brasil ANDROFERT, Clínica de Andrologia e Reprodução Humana, Campinas, SP, Brasil; 2 Universidade Estadual de Campinas Faculdade de Ciências Médicas Departamento de Cirurgia (Disciplina de Urologia) Campinas SP Brasil Departamento de Cirurgia (Disciplina de Urologia), Faculdade de Ciências Médicas, Universidade Estadual de Campinas (UNICAMP), Campinas, SP, Brasil; 3 Aarhus University Department of Clinical Medicine Aarhus Denmark Department of Clinical Medicine, Aarhus University, Aarhus, Denmark

## COMMENT

The scrotal ultrasound (US) examination is a valuable extension of the clinical evaluation of men presenting with infertility ( 1 ). Despite its widespread use, significant heterogeneity persists in how the examination is performed, interpreted, and reported. In this issue of the International Brazilian Journal of Urology, Professor Francesco Lotti provides an expert and meticulously crafted roadmap for urologists to perform scrotal ultrasound with precision and consistency ( 2 ).

### From Routine Imaging to a Structured Diagnostic Tool

In his invited Expert Opinion, "Beyond the Basics: Best Practices in Scrotal Ultrasound for the Infertile Male ( 2 )," Prof. Lotti synthesizes the latest evidence and consensus from leading societies, including the European Academy of Andrology (EAA), the European Society of Urogenital Radiology (ESUR), and the European Association of Urology (EAU). The article delivers an exemplary step-by-step description of the scrotal US examination, highlighting its diagnostic role in evaluating testicular volume, echotexture, vascularization, and the epididymis and vas deferens. Importantly, the paper integrates standard operating procedures (SOPs) and evidence-based reference values derived from healthy, fertile men—an invaluable contribution to the standardization of male infertility workups. It also discusses when the scrotal ultrasound should be combined with transrectal ultrasound examination, which is invaluable for the diagnosis and management of infertility due to ejaculatory duct obstruction ( 3 ).

### Why the Formula Matters: Ellipsoid vs. Lambert

One of the practical pearls emphasized by the author—and deserving special attention—is the recommendation to adopt the ellipsoid formula (length × width × height × 0.52) for calculating testicular volume. This method, endorsed by both EAA and ESUR, correlates more closely with Prader orchidometer estimates and is automatically computed by most US consoles. Historically, the Lambert formula (×0.71) was recommended by radiological societies, but evidence now supports the ellipsoid correction factor of 0.52 for superior accuracy and clinical reproducibility. The shift to the ellipsoid formula thus represents more than a technical adjustment—it signifies the alignment of urologic practice with validated andrology-based standards.

### Technical Precision: Getting the Basics Right

Although the article does not delve deeply into the technical setup of scrotal ultrasonography, it is worth emphasizing a few practical considerations that further enhance the quality and diagnostic yield of the scrotal ultrasound examination. For optimal image resolution, a high-frequency linear transducer (7 MHz or higher) should be used in most cases. In comparison, a lower frequency probe (3-4 MHz) or curved linear transducer (5-7 MHz) may be employed for larger scrotal contents such as hydroceles. The equipment must feature Color and Spectral Doppler, a wide dynamic range, and ideally a trapezoidal imaging mode to enable comprehensive assessment of testicular and epididymal anatomy and perfusion.

A frequency range between 7 and 15 MHz is generally recommended for normal-sized scrotums, ensuring optimal visualization of superficial structures, whereas lower frequencies provide greater tissue penetration when necessary. The trapezoidal imaging feature, available on many modern probes, expands the field of view, facilitating complete visualization of both testes and epididymides. Equipment with a wide dynamic range improves tissue contrast, while Color and Spectral Doppler modes are indispensable for assessing testicular and spermatic cord perfusion. They are also crucial for detecting slow blood flow in conditions such as varicocele or torsion, where Power Doppler often provides greater sensitivity.

Adjustable depth (typically 1–5 cm for scrotal contents) and Doppler frequency settings are essential to optimize image quality. Generous gel application ensures good acoustic coupling, and while elastography can aid in characterizing focal lesions, it remains an optional adjunct rather than a standard requirement.

### Clinical Context Still Rules: The Case of Varicocele

A further highlight is the nuanced discussion of varicocele assessment. While Doppler ultrasound offers superior sensitivity in detecting venous reflux and grading disease severity, treatment decisions must remain anchored in clinical examination, not imaging alone ( 4 , 5 ). This principle—reaffirmed by major international guidelines—safeguards against overdiagnosis and ensures that surgical correction is reserved for clinically significant cases ( 6 , 7 ). Indeed, the surgical repair of clinical varicocele has been associated with improvement in semen parameters, increased rates of natural assisted pregnancies, and reduction in sperm DNA fragmentation rates ( 8 - 14 ). The intervention is indicated for infertile men with clinical varicocele (grades I to III) accompanied by semen abnormalities (concentration, motility, and/or morphology, or DNA fragmentation) or altered biochemical markers (e.g., creatine kinase, reactive oxygen species) ( 1 , 8 , 10 ). The preferred surgical technique is microsurgical subinguinal varicocelectomy due to its high success rate and lower complication rate ( 1 , 10 ).

## CONCLUSIONS

Prof. Lotti, from the University of Florence, Italy, has been instrumental in defining normative scrotal US parameters and advancing the standardization of male genital imaging. As he notes: " *Our goal is to provide a shared language and reproducible framework for scrotal ultrasonography in male infertility. By harmonizing technique and interpretation, we can bridge radiologic precision and clinical relevance, ensuring that every examination truly informs patient care.* "

This Expert Opinion by Prof. Lotti represents a must-read for all urologists and andrologists. It merges scientific rigor with clinical pragmatism and will undoubtedly serve as a reference for training, clinical practice, and research. By advocating standardized methodology and evidence-based interpretation, it sets a new benchmark for quality in male reproductive imaging and strengthens the bridge between diagnostic precision and therapeutic decision-making.

## Data Availability

All data generated or analysed during this study are
